# Calcium-Binding Proteins in the Nervous System during Hibernation: Neuroprotective Strategies in Hypometabolic Conditions?

**DOI:** 10.3390/ijms20092364

**Published:** 2019-05-13

**Authors:** Giacomo Gattoni, Graziella Bernocchi

**Affiliations:** 1 Department of Zoology, University of Cambridge, Downing Street, Cambridge CB2 3EJ, UK; gg456@cam.ac.uk; 2 Former Full Professor of Zoology, Neurogenesis and Comparative Neuromorphology, (Residence address) Viale Matteotti 73, I-27100 Pavia, Italy

**Keywords:** calcium-binding proteins, hibernation, central nervous system, cytoskeleton proteins, neuroprotection, neurodegeneration

## Abstract

Calcium-binding proteins (CBPs) can influence and react to Ca^2+^ transients and modulate the activity of proteins involved in both maintaining homeostatic conditions and protecting cells in harsh environmental conditions. Hibernation is a strategy that evolved in vertebrate and invertebrate species to survive in cold environments; it relies on molecular, cellular, and behavioral adaptations guided by the neuroendocrine system that together ensure unmatched tolerance to hypothermia, hypometabolism, and hypoxia. Therefore, hibernation is a useful model to study molecular neuroprotective adaptations to extreme conditions, and can reveal useful applications to human pathological conditions. In this review, we describe the known changes in Ca^2+^-signaling and the detection and activity of CBPs in the nervous system of vertebrate and invertebrate models during hibernation, focusing on cytosolic Ca^2+^ buffers and calmodulin. Then, we discuss these findings in the context of the neuroprotective and neural plasticity mechanisms in the central nervous system: in particular, those associated with cytoskeletal proteins. Finally, we compare the expression of CBPs in the hibernating nervous system with two different conditions of neurodegeneration, i.e., platinum-induced neurotoxicity and Alzheimer’s disease, to highlight the similarities and differences and demonstrate the potential of hibernation to shed light into part of the molecular mechanisms behind neurodegenerative diseases.

## 1. Introduction

The individual cells of an organism are not isolated in a stable medium, but are immersed in an ever-changing environment influenced by both the activity of surrounding cells and the external conditions in which the organism is living. Changes in the cell’s environment and cell-to-cell interactions can lead to modifications in gene expression, enzyme activity, and ion exchange between the two sides of the plasma membrane, which are processes that must be strictly controlled to avoid damage, cell degeneration, and death. This is particularly true for neurons, which rely on transient changes in ion concentrations for the transmission of electric signals, and therefore need precise molecular mechanisms to restore and maintain the normal electric potential for transmitting new signals and avoiding the alteration of normal cell activities [1,2]. Moreover, the nervous system has very limited regenerative abilities in many animals; therefore, cell loss could lead to irreversible damage at the tissue level and compromised connectivity [3,4].

Calcium (Ca^2+^) has long been recognized as an indispensable ion in neurons, as it is involved in functions as diverse as membrane excitability, signal transduction, neurotransmitters release, synaptic plasticity, cell cycle regulation, cell migration, and axon growing [5,6,7,8]. A highly conserved signaling toolkit controls the level of intracellular Ca^2+^: Ca^2+^ ions enter the cytoplasm through channels on the plasma membrane, or can be released from intracellular stores in the endoplasmic reticulum, Golgi apparatus, and mitochondria; then, through the activity of pumps and ion exchangers, the Ca^2+^ concentration can return to lower steady-state levels [8,9,10]. These mechanisms allow the formation of Ca^2+^ transients during which Ca^2+^ can act directly or indirectly on several pathways by binding to calcium-binding proteins (CBPs), which interpret and respond to the transients [10,11]. 

CBPs have fundamental roles in animal nervous systems: from the regulation of cellular Ca^2+^ concentration to cell signaling, cytoskeletal remodeling, and protein phosphorylation, which are activities that are modulated throughout the entire life cycle (from development to adulthood), both in homeostatic conditions and to react to changes in the external environment [10,12]. In this review, we provide an overview of the known changes in the detection and activity of CBPs that have been studied and reported in the nervous system during hibernation, which is a strategy used by several invertebrate and vertebrate animals to survive in cold conditions. (i). Our primary goal is to focus on four CBPs: the three main cytosolic buffers, i.e., calbindin (CB), parvalbumin (PV), and calretinin (CR), and the most studied Ca^2+^ sensor, calmodulin (CaM), to shed light on their possible involvement in the molecular and cytological adaptations to hypometabolism and hypothermia that hibernators use to prevent freezing while minimizing energy loss in unfavorable conditions. (ii). Secondly, given the widespread role of calcium signaling, it is not surprising that several pathologies have been correlated to alterations of normal calcium homeostasis [13,14,15,16]; since neuroprotective mechanisms have been demonstrated during hibernation, we show that studying calcium regulation in this model could reveal interesting applications to tackle human pathological conditions such as neurodegenerative diseases. (iii). Furthermore, based on our expertise on the effects of cytotoxic drugs on nervous tissues [17,18,19], we try to clarify the alterations in CBPs in the case of the cytological and histological damage of the nervous system, and compare these models of neurodegeneration to the unmatched tolerance observed in the natural condition of hibernation, taking into consideration the relationship between the components of CBPs and the cytoskeleton.

## 2. CBPs in the Central Nervous System

**Figure 1:**
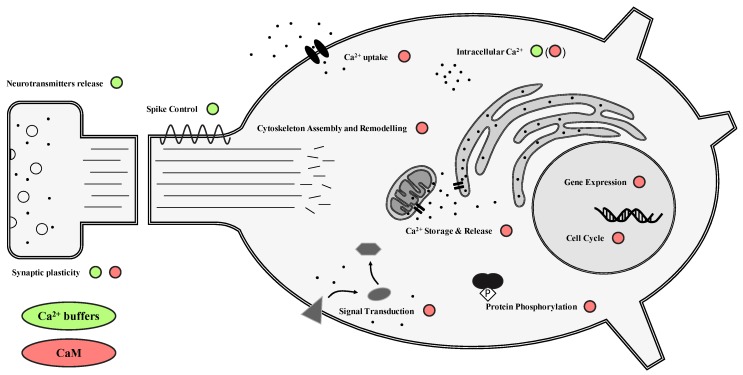
Schematic overview of the functions of buffer and sensor calcium-binding proteins (CBPs) in neurons. Ca^2+^ buffers directly control intracellular Ca^2+^ concentration during Ca^2+^ transients. As a consequence, they can indirectly influence spike duration and intensity, and therefore regulate the release and short-term synaptic plasticity of neurotransmitters. CaM and other Ca^2+^ sensors interact with a wide variety of intracellular proteins, often activating complex cascades of signal transduction through direct interaction or protein phosphorylation. These cascades control gene expression, cell cycle progression, apoptosis, and cytoskeletal remodeling, among other functions. Furthermore, CaM is known to affect the activity of ion channels that control Ca^2+^ uptake and storage, thereby modulating Ca^2+^ transients depending on external and internal signals, and can even act as a buffer at high concentrations. The overall effect on the intracellular Ca^2+^ dynamics of different CBPs influence all aspects of Ca^2+^ signaling, contributing to regulating cell homeostasis and reactions to changes in environmental conditions.

CBPs are a large class of proteins involved in managing and reacting to Ca^2+^ transients in cells. More than 200 proteins with the capacity to bind Ca^2+^ ions have been identified with widespread roles that span from gene expression control to synaptic plasticity, indicating their fundamental importance for the survival of the cell [20,21] (Figure 1). These molecules can essentially be divided into two categories: buffer proteins exert a fine control over the calcium concentration within the cell, while sensor proteins can interact with a wide variety of specific targets depending on the information received through Ca^2+^ signaling [20,22].

### 2.1. Calcium Buffers

Calcium buffers are traditionally described as molecules that can bind calcium with different affinity, but do not exhibit substantial conformational changes, and do not directly influence the activity of other macromolecules. Their primary role is to control the free Ca^2+^ concentration in the cell and modulate Ca^2+^ signals both in terms of spatial localization and temporal duration, ensuring that they are correctly “interpreted” by the cell. Cytosolic calcium buffers can quickly diffuse and therefore determine the mean concentration of Ca^2+^ in the whole cytoplasm; they can also act in concert with other CBPs in precise locations where a finer or quicker control of Ca^2+^ concentration is needed [20,23]. Different CBP buffers exert the same function, but importantly, the number of Ca^2+^-binding sites, the affinity for the ion, and consequently the binding speed can be widely different [22]. In homeostatic conditions, when the intracellular Ca^2+^ concentration is between 20–100 nM, these proteins are in a Ca^2+^ free state, and only during Ca^2+^ transients, when the Ca^2+^ concentration increases by at least one order of magnitude, do the buffers exert their role and start binding the ions [22,23]. The nervous system is particularly enriched in Ca^2+^ buffers, and different subpopulations of neurons express different combinations of CBPs, highlighting the need for a fine control of electrical and synaptic activity. Interestingly, knockout experiments performed on mice, in which one or a combination of Ca^2+^ buffers are inactivated constitutively or in specific cell types using a variety of transgenesis tools, shows that if a CBP is lost, there is no increase in the expression of other types [24]. This indicates that in normal conditions, the expression of many CBPs is highly repressed at the transcriptional level, and suggests that each may have an important and irreplaceable function in the cell. Moreover, this has allowed the use of these proteins as immunohistochemical markers to identify cell types in different areas of the brain, helping to resolve the architecture of the cerebral and cerebellar cortex [25,26,27]. Here, we briefly describe the nervous system localization and function in mammals of the three most studied cytosolic CBPs.

Calbindin D-28k. Calbindin (CB) is a 28-kDa protein with six EF-hand Ca^2+^-binding domains, but only four are active and have rapid binding kinetics. In the brain, it can be found mainly in populations of γ-aminobutyric acid (GABA)-containing interneurons scattered throughout cortical layers but concentrated in supragranular layers; positive interneurons can also be identified in the hippocampus, where granule cells of the dentate gyrus and some CA1 pyramidal neurons are also immunoreactive to this protein, and in the hypothalamus [28,29,30,31]. In the cerebellum, CB is specifically and strongly expressed in Purkinje cells, and in some mammals, in Golgi cells [24,26]. The kinetic properties of CB lead to rapid saturation during Ca^2+^ transients at the pre-synaptic site, followed by a transient decrease in binding capacity that has a role in synaptic facilitation through a mechanism called “facilitation by buffer saturation” [23]. This means that just by controlling the ions’ activity near the synapse Ca^2+^ buffers can have a role in synaptic plasticity. Studies on the null-mutant CB^–/–^ mice have shown that the absence of this protein does not have any developmental or morphological effect, but the impairment of motor coordination has been identified and ascribed to Purkinje cells’ control of motor behavior [24,32]. Interestingly, although no role for calbindin in cell signaling has been identified, this molecule has been shown to bind to several cell components, including the cytoskeleton, likely leading to a more precise localization within the cytoplasm [32,33].

Calretinin. Calretinin (CR) is a less studied, 31-kDa protein with five active Ca^2+^ binding domains that is distributed in a distinct but smaller population of cortical interneurons of the cerebral cortex, in some neurons of the thalamus, and in the granule cells and unipolar brush cells of the cerebellum [26,30,34,35]. CR^-/-^ mice obtained through homologous recombination had impaired long-term potentiation in the hippocampus, while in the cerebellum, the alteration of granule cell excitability following CR depletion also led to altered CB activity and Ca^2+^ homeostasis in Purkinje cells, resulting in a mild motor coordination impairment that is less pronounced than the one observed for CB knockout [24,32]. Recent studies have indicated the implication of CR in several pathways, and therefore an additional role as a Ca^2+^ sensor [35].

Parvalbumin. Parvalbumin (PV) was the first discovered EF-hand-containing protein, with three mixed Ca^2+^/Mg^2+^-binding sites. In the cerebral cortex, PV is expressed in GABAergic subpopulations that do not contain CB, including some stellate, basket, and chandelier cells, distributed in all layers except for layer one [27,30,36,37]. In the hippocampus, basket cells and other interneurons are labeled by anti-PV antibodies throughout all areas [25,38]. In the cerebellum, PV is expressed in Purkinje, stellate, and basket cells and, in humans, it is expressed in some Golgi cells [24,26,39]. The slow kinetics of Ca^2+^ binding by PV and the agonistic relationship with Mg^2+^ ions leads to a “slow-onset” Ca^2+^-buffering action by PV. While the rise in Ca^2+^ concentration is not affected by this protein, the late phase of decay at the end of the transient is prolonged, leading to a biexponential decay and a delayed release of neurotransmitters in some synapses, as discovered in cerebellar interneurons [22,23,32]. PV knockouts in mice strains have been repeatedly obtained for 20 years, first through homologous recombination and more recently using *cre/lox* recombination combined with tetracyclin-dependent or tamoxifen-dependent techniques to create conditional knockouts in specific cell types [32]. These studies confirmed the role of PV as a “slow” CBP: in Purkinje cells, the absence of the PV does not impair the initial inhibitory post-synaptic currents; rather, it modulates synaptic plasticity in the short-term period [24]. A similar conclusion was reached on the role of PV in hippocampal interneurons [32]. 

### 2.2. Calcium Sensors

Controlling Ca^2+^ concentration buffers can have indirect effects on neurons’ excitability and synaptic plasticity; on the other hand, Ca^2+^ sensors can bind calcium and undergo conformational changes that reveal or activate binding sites for other proteins, and therefore directly regulate their functions. Moreover, if present in high concentrations, calcium sensors can also act as calcium buffers [40]. Several members of this class have been identified in the nervous system, including a large class of neuronal calcium sensors (NCS) [41], but by far the best studied and most important Ca^2+^ sensor is the ubiquitous protein calmodulin (CaM). This small protein is conserved in all eukaryotes and consists of two domains, each with two EF-hand Ca^2+^-binding sites connected by a linker that ensures a great structural plasticity that is the secret to calmodulin’s impressive ability to bind to a large number of targets depending on the concentration of Ca^2+^ ions [42,43]. It is hard to underestimate the importance and the variety of calmodulin functions in the cell: dozens of targets have been identified, including enzymes, channels, pumps, transcription factors, and structural proteins [44,45,46,47,48,49,50]. One of the main ways in which calmodulin modulates cells’ activities in response to Ca^2+^ stimuli is by controlling protein phosphorylation though the regulation of kinases and proteases [51,52]. If a kinase is in an autoinhibited state, the binding with Ca^2+^/CaM can release the inhibition and activate different processes, including signal transduction, gene expression, and cytoskeletal remodeling [52,53]. Similarly, CaM can bind and regulate the activity of Ca^2+^ channels or pumps, thereby influencing the intensity and/or duration of calcium transients [8,23,54]. 

Given their roles in the cell and their use as neuron markers, these CBPs have often been exploited to identify changes in the brain in pathological or stress conditions, which in many cases lead to significant alterations in the number of positive neurons and the amount of intracellular CBPs [55,56,57,58]. In the next sections of this review, we focus on the molecular and histochemical changes in CBPs activity in hibernation, which is a model of cell tolerance to hypometabolic and hypothermic conditions.

## 3. Hibernation: Who, How, and Why is it Interesting?

All organisms are influenced throughout their life cycle by biotic and abiotic factors that are extremely variable in space and time, and several strategies have evolved to react to adverse environmental changes. Hibernation is one of the most widespread and best studied strategies to cope with low temperatures: during winter in temperate and seasonal areas, numerous animals can strongly reduce metabolic activity and temporarily suspend movement, nutrition, and growth [59,60,61]. All hibernating species require the integration of molecular, cellular, and behavioral adaptations that together orchestrate the entrance into the dormancy state and ensure that tissues are protected from damage [62,63,64,65].

Among vertebrates, hibernation has been mostly studied in mammals, for which we now have a large body of literature describing adaptations in cell gene expression, biochemistry, and physiology [66,67,68,69,70,71]. While the precise endocrine signals that induce hibernation are still unknown, the hypothalamus is thought to play a key role in decreasing metabolic rate and inhibiting heat production, leading to a gradual decline in body temperature [72]. The hypothalamus remains active during hibernation when compared to the rest of the brain, and it seems to maintain thermoregulatory control to avoid freezing [73]. Mammal hibernation is generally characterized by a succession of torpor bouts and arousal periods during which temperature oscillates between the torpid level and normothermia, and whose role and significance are still obscure [72,74,75]. During torpor, cells are usually in hypothermic and hypoxic conditions, but at arousal, no widespread damage can be detected at the cellular and tissue level [76,77]. Moreover, hibernating species show resistance to these conditions even when they are not hibernating [78,79,80]. This suggests that specific cellular adaptations in hibernating species ensure protection from damage, but these mechanisms have only been partially explained. For example, tolerance to hypoxia seems to arise from a combination of higher hemoglobin affinity for oxygen, and in the brain, increased antioxidants activity, regulation of the immune system, and alterations in ion channels’ activity [78,81]. Strikingly, the heart and skeletal muscles of hibernating ground squirrels showed the preservation of intracellular Ca^2+^ concentration [82,83]. This homeostasis is maintained even in harsh conditions through a reduction of Ca^2+^ influx by the suppression of Ca^2+^ channels and by an increased uptake by the endoplasmic reticulum, in which a hibernator-specific isoform of calsequestrin, a sarcoplasmic CBP, facilitates the uptake and storage of the ion [84,85].

Endotherms are not the only animals that can hibernate: several vertebrate and invertebrate ectotherms have evolved strategies to avoid or tolerate freezing temperatures in hypometabolic conditions [86,87]. Among vertebrate ectotherms, many frogs possess remarkable tolerance to freezing, and can therefore live in extremely cold environments [88]. Invertebrates such as pulmonate gastropods, despite being adapted to life on land, are still susceptible to dehydration and freezing in winter. To avoid this, some species of snail hibernate. The main environmental factor that triggers hibernation in these molluscs is the photoperiod, although temperature and humidity also play a role [89]. In this state, the snails enclose themselves into their shells and avoid the formation of extracellular ice by concentrating the hemolymph, eliminating ice nucleating agents, and accumulating low molecular weight anti-freezing molecules such as glycogen [90,91,92,93]. The neuroendocrine control of hibernation entrance in molluscs is still unknown, but the large size of their neurons and the simple and well-known structure of their neural circuits make them suitable models for immunohistochemical and physiological studies, as the subcellular localization of molecules can often be inferred and the electrical activity of neurons can be precisely tracked and controlled [94,95,96], providing a unique opportunity to study the cytological basis of behavior. For example, several molecules have been shown to change their localization or expression level during hibernation when compared to activity, including bioactive peptides, neurotransmitters, and ion channels [97,98,99,100,101,102,103,104].

This brief overview shows that hibernation has attracted attention for four main reasons:

- It is a convenient and efficient model to study reaction and adaptation to extreme conditions at different levels of organization, from behavior down to the genetic control and the changes in molecular interactions.
- Hibernating animals show unmatched tolerance to these conditions thanks to protective mechanisms; this is especially true for the nervous system, which requires large amounts of energy to be maintained.
- It is an interesting case of convergent evolution, as these strategies independently evolved several times in distantly related phyla and closely related groups of mammals. By comparing similarities and differences, general properties of cell resistance could be discovered [81,105].
- Understanding these properties could have direct applications to human health, as similar protective mechanisms could be harnessed to cure or prevent human pathologies [106,107,108].

## 4. CBPs in the Hibernating Nervous System: A Role in Neuroprotection and Plasticity?

As the central nervous system (CNS) controls both fast responses to external stimuli and, through the regulation of hormone production and release, slower systemic reactions guided by endocrine organs, it has to influence any type of regulated change in animal physiology and behavior to ensure synchronization among different processes without damaging the organism. In hibernating animals, the nervous system needs to remain at least partially active to guide entrance into torpor and arousal, but at the same time, it requires neuroprotective strategies to survive in hypometabolic and hypothermic conditions in the same way as the rest of the organism [73]. Moreover, as several activities are suspended or greatly reduced, the connectivity of the CNS is expected to undergo passive and active remodeling, providing a unique opportunity to analyze molecular and functional aspects of neural plasticity. 

### 4.1. Ca^2+^ and CBPs

Numerous protective mechanisms have been identified in the CNS of mammals during hibernation [109]. The hypothalamus is a key regulator of torpor entrance together with a set of hibernation-specific proteins (HP) that are thought to induce permissive hormonal signals to prepare neurons for hypothermia and reduced activity [110,111]. The neuromodulator adenosine is involved in regulating the onset of torpor, and it has been experimentally shown that exogenous application in the CNS can induce hypothermia [111,112]. Interestingly, the injection of Ca^2+^ in the brain of ground squirrels and hamsters triggered deep hypothermia, suggesting that it may also be involved in torpor entrance by contributing to establish a new body temperature set point [113,114]. During torpor, adaptations such as inflammatory activation, antioxidant production, and apoptosis avoidance ensure an unmatched tolerance at the cellular and system levels [109,115,116]. These adaptations are paralleled by significant changes in gene expression, which are thought to occur through differential control by transcription factors and chromatin remodeling [84,117,118,119]. These changes involve among other functions metabolism, response to hypoxia, DNA repair, cytoskeletal remodeling, and Ca^2+^ signaling [120,121,122].

Information on Ca^2+^ concentration in mammal neurons during hibernation is scarce: only one study showed that in ground squirrels, resting Ca^2+^ concentration and Ca^2+^ accumulation in synaptosomes are both reduced during hibernation [123]. However, insights on Ca^2+^ signaling can also be deduced by studying the wide variety of molecules that interact with this ion. In particular, several works have highlighted changes in the detection of CBPs between the active and inactive periods in numerous species. We have summarized the results in Table 1. The majority of these studies have identified and quantified different CBPs in non-model species through Western blot and immunohistochemistry, comparing activity and hibernation periods both in terms of number of stained cells and the intensity of immunoreactivity. In more recent years, gene expression and proteomic analyses have opened the possibility for a more comprehensive understanding of life-cycle changes, including those associated with hibernation [121,124,125]. 

Regarding our experience on CBPs in hibernating vertebrate models, specific changes were found in the CNS, with different brain areas showing different degrees of variation in CBP content, supporting the idea that each part of the brain has distinct functions and activity levels during hibernation. Immunoreactivity for buffer and sensor CBPs decreased during hibernation in the cerebellum of hedgehogs and frogs [126,127]. In particular, Purkinje cells showed drastically decreased CB and PV immunoreactivity after a long hibernation period in the hedgehog, which was paralleled by a decrease in PV and CR positivity in the molecular and the internal granular layer [127]. In the frog cerebellum, Purkinje and stellate cells showed a decreased positivity for CaM [126]. Similarly, in the cortex of ground squirrels, the expression of the genes encoding CaM and CR is decreased in torpid animals compared to active ones, but no change in CB or PV expression could be detected [128]. This could mean that protein levels are controlled at the post-transcriptional level, and highlights the importance of comparing immunohistochemical and gene expression data: indeed, it has been shown that protein synthesis is altered in the inactive phase [129]. On the other hand, the expression of buffer protein genes in the hypothalamus remained the same throughout the annual cycle of the thirteen-lined ground squirrel, and only a small, non-significant decrease was observed for CaM, in accordance with the idea that this area remains active throughout torpor, controlling body temperature and arousal [128]. In this context, buffer proteins in the hypothalamus would guide Ca^2+^ transients similarly to euthermic months, avoiding alterations of neurotransmitters release and synaptic rearrangements that could delay or compromise neuronal activity.

Moving to invertebrates, the CNS of the garden snail *Cornu aspersum* is an excellent model to study cytological and histological changes during the annual cycle due to the large size of its neurons. It consists of a pair of cerebral ganglia connected with anterior buccal ganglia, and a large suboesophageal ganglion that sends projections to different organs in the visceral sac [130,131]. The comparison of free Ca^2+^ ions using ion-sensitive microelectrodes between active and hibernating neurons showed a significant increase during the inactive period [132]. Persistently high intracellular Ca^2+^ concentration can potentially lead to cell damage or apoptosis, but aroused animals show no signs of degeneration, indicating the presence of regulated protective mechanisms. The expression and activity of sodium channels was shown to significantly decrease during hibernation [99], and Ca^2+^-ATPase-like immunoreactivity was lower in hibernating specimens compared to active ones [95], suggesting a differential regulation of ion exchange between the two sides of the cell membrane and a decrease in Ca^2+^ removal that could partially explain the increased Ca^2+^ concentration in hibernating neurons. We recently showed that CaM-like immunoreactivity significantly increases during hibernation in the cerebral ganglion, and that the level of PV-like is unaltered throughout the entire annual cycle [133]. The high level of these CBPs during hibernation could contrast and maintain the higher intracellular Ca^2+^ concentration, avoiding cytotoxic effects, as high concentrations of Ca^2+^ sensors can have a buffering effect. 

**Figure 2:**
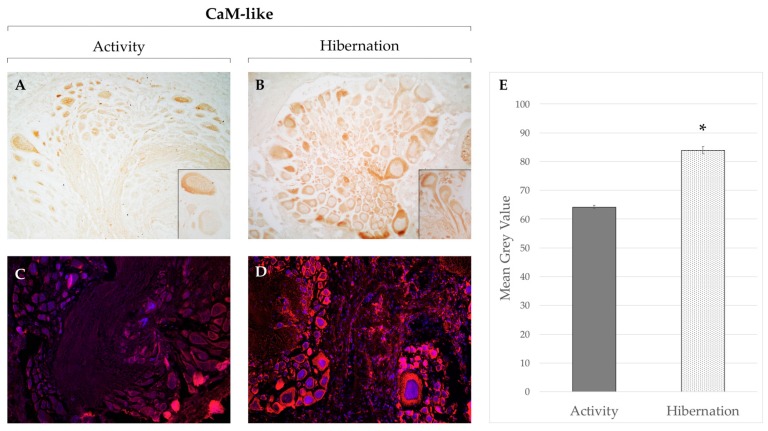
Immunohistochemistry with light (**A**,**B**) and fluorescence (**C**,**D**) microscopy for the Ca^2+^-binding protein calmodulin-like (CaM-like) in the suboesophageal ganglia of *Cornu aspersum* during activity and hibernation. In active snails, pale CaM-like immunoreactivity was detected across the ganglion (**A**,**C**) with only few sparse cells showing strong staining in the cytoplasm (**A**, insert). Hibernating suboesophageal ganglia showed a stronger and more diffuse positivity to CaM-like in all panels evaluated (**B**,**D**). The signal could be detected in both the somata and axons of most neurons (**B**, insert). Quantitative analysis on immunofluorescence labeling demonstrated that the change between activity and hibernation is highly significant (asterisk: Student’s *t* test, *p* value < 0.01) (**E**). Experiments were carried out as described in [133]. Magnification: 10× (A–D).

To test whether the increased CaM-like positivity is specific to the cerebral ganglion, here we have further compared CaM-like immunoreactivity in the suboesophageal ganglion of active and hibernating *C. aspersum* combining chromogenic and fluorescent immunohistochemistry (Figure 2). While only few cells showed positivity for this marker in the cytoplasm in awake snails (Figure 2A, insert), most neurons were strongly immunoreactive during hibernation (Figure 2B,D). By quantifying immunofluorescence intensity using the ImageJ particle analysis tool (ImageJ 1.51 s; NIH, Bethesda, MA, USA) as described previously [133], we showed that the observed changes between activity and hibernation are highly significant (Figure 2E), indicating that CaM plays a key role in neuron survival in the entire nervous system by buffering Ca^2+^ concentration or by binding to other effector proteins.

In accordance with this hypothesis, the activity of numerous molecules has been shown to change during hibernation in both vertebrate and invertebrate models. Ca^2+^-dependent kinases and phosphatases are particularly interesting in this context for their potential to respond to variations in Ca^2+^ concentration. Arendt et al. monitored the activity of several kinases during torpor in Arctic ground squirrels [140], detecting shifts in both directions: glycogen synthase kinase (GSK-3ß), extracellular regulated protein kinase 2 (ERK2), and stress-activated protein kinase/Jun-amino-terminal kinase (SAPK/JNK) activity decreased, while protein kinase A (PKA) and ERK1 activity increased during hibernation. Unfortunately, despite their essential role in the cell and their reported association with cold resistance [141,142], Ca^2+^/CaM-dependent protein kinases have not been considered in these studies, and a deeper understanding of their activity is needed to conclusively interpret the distribution of this Ca^2+^ sensor. Gene expression analysis showed a decrease in CaMKII gene expression in the cortex of torpid ground squirrels, while no difference could be detected in the hypothalamus, suggesting that in inactive areas of the mammalian brain, CaM-dependent phosphorylation might be downregulated following CaM decrease [128]. Other studies have compared the expression and activity of different phosphatases: protein phosphatase 2A (PP2A), one of the most important enzymes involved in dephosphorylation, showed a decreased gene expression and activity in different independent works [135,143,144,145], while other enzymes such as protein phosphatase 1 (PP1) and protein phosphatase 2C (PP2C) had a significant increase in activity levels during hibernation [143]. 

### 4.2. CBPs and Cytoskeleton Components

Taken together, these results show that specific changes in Ca^2+^ signaling, CBP localization, and protein phosphorylation are characteristic of the hibernating phase in both vertebrates and invertebrates. The differences seen between hibernating species are likely a result of the independent evolution of this strategy in different lineages, but the significance of these cytological modifications strongly points to a functional role in allowing neuron survival in hypometabolic and hypothermic conditions by changing the interactions among different pathways. At the cell level, these molecular adaptations are paralleled by visible morphological remodeling that has been ascribed to both plastic and neuroprotective strategies. In fact, during hibernation in mammals, a substantial but reversible modification of neural connectivity has been discovered in the hippocampus [146,147] and later in other areas of the encephalon [148], which is characterized by a decrease in cell body area, branching complexity, and spine density, and by an alteration of synaptic protein content [136,149,150]. This retraction is readily reversed during arousal periods, and does not seem to be associated with memory loss [139,151,152,153]. Such an impressive phenomenon requires a controlled modification of the cytoskeleton, which will then translate into cell-shape change. Accordingly, significant changes in the gene expression and protein localization of cytoskeletal components between activity and hibernation have been discovered over the years, and are often associated with Ca^2+^-interacting molecules for their recognized roles in the control of cytoskeletal assembly [122,154] (Table 1). 

Microtubules are among the main constituents of the cytoskeleton and, together with defining the cell shape, they have a primary role in molecular transport and synaptic transmission in neurons. The dynamic balance between assembly and disassembly is controlled by microtubule associated proteins (MAPs) which, depending on their phosphorylation state, can bind and stabilize tubulin subunits [155,156,157]. Microtubule-associated protein 2 (MAP-2) immunoreactivity was shown to decrease in the cerebellum of the European hedgehog and European ground squirrels during hibernation [127,134], and to transfer from a dendritic/synaptic to a cytoplasmic localization in mammals and frogs [126,136,158], following the decrease of synaptic complexity. As the expression of the MAP-2 gene does not change across the brain, these results can be ascribed to a reduced translation with the aim of increasing neuroplasticity and at the same time storing the protein in the cytoplasm so that it can be readily used after arousal [128,136]. Using an anti-MAP-2 antibody on snail neurons, we recently noted an increase in MAP-2-like immunoreactivity in the soma of cerebral ganglia neurons of *C. aspersum* during hibernation that may improve cytoskeletal stability [133], while in the neuropil, a decreased immunopositivity was discovered in a previous work [95], indicating similar modifications in the intracellular localization of MAPs in both vertebrate and invertebrate models. Tau is another MAP that can stabilize microtubules in its phosphate-free form, whereas phosphorylation by several kinases, including Ca^2+^/CaM-dependent kinases, causes its detachment from microtubules and an accumulation in its free-form in the cytoplasm [159,160]. During hibernation, a highly significant increase in tau phosphorylation was observed in both mammals and molluscs, despite the differences in the physiology between the different animals [133,134,137,158,161]. In fact, high phosphorylated tau (P-Tau) has been found in neurons of obligate and optional hibernators with both continuous dormancy or torpor-arousal cycles. While in humans hyperphosphorylated tau is associated with neurodegenerative disorders collectively called Tauopathies, P-Tau levels in hibernators quickly return to euthermic levels after arousal [134,135,162]; the implications of this interesting phenomenon will be discussed in the following section.

CaM is implicated in the regulation of MAPs activity through two complementary mechanisms: firstly, this CBP can control the activity of kinases and phosphatases that act on these cytoskeletal proteins, as exemplified by Ca^2+^/CaM-dependent kinases’ control of tau phosphorylation [160,163,164,165]; secondly, CaM can directly bind to both MAP-2 and tau with a flip-flop mechanism by which CaM binding prevents the association of MAPs and microtubules in a Ca^2+^-dependent manner, thereby destabilizing microtubules [166,167,168]. Interestingly, in the neurons of *C. aspersum*, both CaM- and P-Tau-like immunoreactivity showed the co-localization of strongly immunopositive masses during hibernation, suggesting a stage-specific interaction that could either be direct or involve the mediation of kinases, which may increase the levels of P-Tau and result in decreased binding to microtubules [133]. In mammals, the decrease of PP2A activity, one of the main tau phosphatases, is greater than the decrease in the activity of tau kinases, likely leading to a net increase in the level of tau phosphorylation [158]. With the reversal of kinase and phosphatase activity and CBP levels at arousal, tau proteins are then dephosphorylated to normal levels.

Neurofilaments are nervous system-specific class IV intermediate filaments and the main constituents of neuron cytoskeleton, with roles that span from development and shape to transport and plasticity [169]. The phosphorylation of neurofilament subunits is an essential process for their activity: for example, the heavy subunit of neurofilaments (NF-H) is found in its phosphate-free form in the somata, where polymerization is inhibited, while phosphorylation progressively increases along the axons where the subunits are added to the bigger neurofilament protein [170]. 

**Figure 3:**
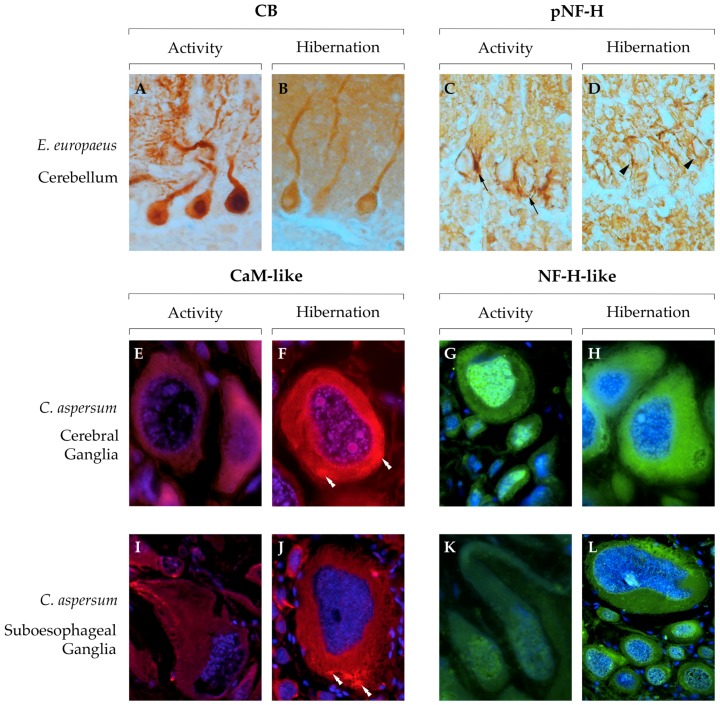
Comparison of immunohistochemistry staining for CBPs and NF-H between activity and hibernation in vertebrate (European hedgehog, **A**–**D**) and invertebrate (garden snail, **E–L**) models. In the cerebellum of active hedgehogs, the cytoplasm and dendritic tree of Purkinje neurons showed strong CB labeling (**A**), while a visible decrease was detected after a long period of hibernation (**B**). NF-H immunopositivity was present in axons and terminals around Purkinje cells somata during activity (**C**, arrows), and strongly decreased after a long period of hibernation, when it was observed only in few terminals (**D**, arrowheads). In the cerebral (**E**–**H**) and suboesophageal (**I**–**L**) ganglia of the garden snail, a significant increase in the number of positive neurons and intensity of immunoreactivity for CaM-like during hibernation (**F**,**J**) was accompanied by an increased recognition of epitopes by anti-NF-H antibodies (**H**,**L**) with respect to activity (**E**, **I** and **G**, **K**, respectively). Throughout the snail CNS, several neurons showed hibernation-specific aggregates of CaM-like in the cytoplasm (**F**, **J**, double arrowheads). Experiments were carried out as described in [127,133]. Magnification: 40× (**A–D**, **I–L**); 60× (**G**); 100×(**E**,**F**,**H**).

The total mRNA and protein levels of NF-H in mammals increase in the hibernating cortex of the 13-lined ground squirrel [122,128]; however, immunohistochemical data show that in both mammals and amphibians, the amount of NF-H phosphorylation decreases during hibernation, implying an alteration in the dynamic growth of neurofilaments [126,127]. Instead, in the snail, immunohistochemical reactions using anti-NF-H antibodies showed that both total and phosphorylated NF-H-like immunoreactivity increases in the hibernating cerebral ganglia [95,133], suggesting that the accumulation of neurofilaments in the somata might be a convergent strategy to increase cellular stability and allow storage of the protein to be re-used after arousal. On the other hand, the level of phosphorylation differs in the independently-evolved strategies; the increase in the epitopes recognized by the phosphorylated anti-NF-H antibody in snails could have the function of protecting neurofilaments from the action of proteases [171], while the decrease in pNF-H in vertebrates probably follows the reduced need for axonal transport and stability. Accordingly, we show here that the total NF-H-like immunoreactivity increases in the hibernating suboesophageal ganglia of *C. aspersum* as part of a general neuroprotective strategy. Interestingly, the increase in NF-H-like immunoreactivity is correlated with an increase in CaM-like immunoreactivity in snails, while in vertebrates, the decrease in NF-H phosphorylation is associated with a decrease in CBPs detection, as demonstrated by the hedgehog (Figure 3) and frog cerebellum, supporting the link between CBPs and cytoskeletal proteins. In summary, these results indicate that CBPs play an important role in the nervous system in the process of hibernation as demonstrated by the extensive changes in their distribution when compared to the active period of the annual cycle. Moreover, these changes and the correlation with other molecules involved in cell reorganization and resistance, such as cytoskeletal proteins, offer a unique opportunity to formulate testable hypotheses on the mechanisms of neuroprotection and neural plasticity.

## 5. Hibernation and Translational Medicine: the Case of Neurodegeneration

The exceptional tolerance to hypometabolic, hypothermic, and hypoxic conditions observed in hibernating animals has attracted a lot of attention in the past two decades due to the possible applications to human health. Since molecular pathways controlling different functions are highly conserved in mammals, understanding how hibernating cells resist to harsh conditions could give us valuable information on how to recreate those protective strategies in humans. The best example is represented by the impressive tolerance to ischemic conditions in torpid animals, which has been extensively analyzed and reviewed to look for possible strategies to reduce brain damage [57,172,173]. Similarly, hibernation has been studied for its potential to improve organ transplant techniques, particularly with respect to controlled hypothermia that can trigger short-term protective mechanisms [108,174,175]. These studies make use of in vitro and ex vivo models and focus on molecules that induce some of the phenotypes observed during hibernation, evaluating cytological and morphological modifications together with overall tissue damage. The synthetic peptide [D-Ala2, D-Leu5]-enkephalin (DADLE) was shown to induce hibernation when injected in active ground squirrels [176], protect against the negative effects of ischemia [175,177], and drastically increase the survival time of explanted organ preparations [178]. At the cell level, DADLE interacts with delta opioid receptors and reduces proliferation and RNA transcription, but the mechanisms of action are still poorly understood [177,179]. It has been speculated that in neurons, the activation of delta opioid receptors could reduce Ca^2+^ dysregulation and inhibit glutamate release preserving ionic homeostasis and avoiding apoptosis and excitotoxicity [175]. Strikingly, another molecule extracted from hibernating mammal’s blood that can induce hibernation in summer-active ground squirrels, which is called the hibernation induction trigger (HIT), was suggested to bind to delta opioid receptors or induce release of opioid peptides [179]. This example demonstrates the potential of these models to apply the information obtained in hibernating animals to human health, and at the same time help unravel some of the physiological and biochemical mechanisms that are characteristic of hibernation and their relation to more general strategies of cell resistance. 

An interesting aspect of hibernation is the absence of extensive neurodegeneration: several complementary mechanisms are used by torpid mammals to avoid apoptosis, and no alterations in tissue organization and connectivity can be detected at arousal [115,180]. Moreover, in the frog’s brain, the increased cell death observed during hibernation is balanced by increased cell proliferation in the corresponding ventricular areas [181]. The lack of severe cellular damage can also be inferred by comparing hibernation with models of cytotoxicity and neurodegeneration. Neurotoxicity associated with the treatment of platinum compounds has been thoroughly studied for the wide use of these substances as chemotherapeutic agents [182,183,184,185]. Here, we focus briefly on experimental works carried out by our research group at the Department of Biology and Biotechnology (formerly Animal Biology) of the University of Pavia from 1986 to the present, which have revealed alterations in the immunopositivity for CBPs in developing and adult rats exposed to two platinum compounds, cisplatin (CisPt) and [Pt(*O,O′*-acac)(γ-acac)(DMS)] (PtAcacDMS) (Table 2). These studies have highlighted a dynamic decrease in two buffer CBPs, CB and PV, following treatment with both compounds in the hippocampus and cerebellum, and also detected a less severe damage by PtAcacDMS compared to CisPt, demonstrating that CBPs can help discriminate between compounds by providing a reliable estimation of neuronal damage [19,39,186,187,188]. Interestingly, contrary to what was observed in hibernating animals, there is no correlation between changes in CBPs and cytoskeletal detection, as CB immunolabelling decreases while NF-H phosphorylation increases following CisPt treatment [18,188], and is likely responsible for the marked morphological signs of degeneration observed in Purkinje cells of the developing rat cerebellum [189]. This indicates that the two processes rely on different pathways, and suggests that the changes in CBP detection during hibernation are not a sign of neurodegeneration.

In this context, we find it of particular interest to compare these results with a pathological condition such as Alzheimer’s disease (AD), which is the most widespread and costly neurodegenerative disease in the world, accounting for more than 50% cases of dementia [190]. Despite the great progress in understanding the cellular and molecular aspects of this disorder, its causes are still obscure, and it remains one of the biggest health problems that lack a successful cure. Two of the well-known hallmarks of AD are the formation of ß-amyloid plaques in the extracellular space and the accumulation of hyperphosphorylated tau in the form of neurofibrillary tangles (NFTs) in the neuron cytoplasm, but the exact significance of these two features in the pathogenesis is still unclear [191,192]. Among the various pathways involved in AD, the Calcium Hypothesis of Alzheimer’s disease and brain ageing, which was formulated in 1994, states that long-lasting alterations in Ca^2+^ homeostasis and Ca^2+^ signaling play a pivotal role in AD and other disfunctions associated with aging by contributing to all the mechanisms underlying brain damage, including but not limited to amyloid plaques and NFTs [16,193,194]. In accordance with this scenario, studies focusing on CBPs have revealed extensive alterations associated with both normal aging and AD, which are summarized in Table 3. CB immunoreactivity decreases with aging together with the number of positive neurons in various areas of the brain, indicating a general susceptibility to the damage of brain interneurons, which is reflected by the severe decrease associated with AD in humans as well as mouse models of the disease. On the other hand, PV localization is not negatively affected by age, and some studies even report an increase in immunopositivity during ageing. However, AD causes a significant decrease in the number of PV interneurons, indicating the specific neurodegeneration associated with the disorder. Interestingly, some studies have hypothesized a possible neuroprotective role for these two buffer proteins, as CB- and PV-positive GABAergic interneurons are less sensitive to the formation of NFTs in AD [195]. CaM response to aging is much more variable in the CNS depending on the area, but in humans, a decrease in CaM immunoreactivity can be observed throughout the cortex in AD, indicating alterations in Ca^2+^ signaling [196,197].

Regarding the state of cytoskeletal proteins, almost 20 years ago, Arendt et al. discovered that tau hyperphosphorylation, one of the hallmarks of AD, was also present during hibernation in ground squirrels, but was not associated with the formation of NFTs, and fully reversed back to a steady-state level quickly after arousal [134]. Subsequent studies demonstrated that this is a common feature of obligate and permissive mammal hibernators, and is the result of passive temperature-dependent and active hibernation-specific mechanisms [137,161]. The phosphorylation of tau in these animals is interpreted as a mechanism to destabilize microtubules, as P-Tau is unable to bind to tubulin; therefore, it is considered a neuroprotective strategy to improve neural plasticity [158]. Moreover, it has been shown that a moderate and controlled phosphorylation of tau may actually protect cells against apoptosis [198,199]. The phosphorylation of tau is under the control of different kinases, including Ca^2+^/CaM-dependent protein kinases, and can therefore be influenced by changes in Ca^2+^ concentration and CBP distribution [58,160]. In a recent publication, we observed increased P-Tau-like immunoreactivity in the cerebral ganglia of *C. aspersum* in the form of strongly immunoreactive masses distributed in the cytoplasm of neurons, suggesting that tau phosphorylation could be an ancient neuroprotective strategy exploited by cells to survive in adverse conditions that is independently co-opted in different lineages [133]. These aggregations specifically co-localized with CaM-like epitopes during the hibernation phase, hinting at a Ca^2+^-dependent control of tau activity. 

Tau phosphorylation is known to increase following hypothermia and hypometabolism [217,218]. Intriguingly, the analysis of AD symptoms showed that neurons are subject to hypometabolic conditions early during the course of the disease [219,220,221,222]. These findings support the hypothesis that tau phosphorylation may initially be a protective or compensatory mechanism in the pathogenesis of AD to suppress apoptosis and imbalance in neural transmission and Ca^2+^-signaling that follow hypometabolism, whose causes are still unclear. In this scenario, neurons that express buffer CBPs are initially protected against abrupt changes in Ca^2+^ concentration, and therefore show less accumulation of P-Tau [138]. However, in hibernating mammals, tau phosphorylation reverts to a normal state following arousal, avoiding cytotoxic effects; it has been proposed that the need to return to low levels of P-Tau could be one of the main constraints that explain the cycles of torpor and arousal observed in most mammals [158]. In fact, the energy required for re-warming the body during these periodic arousal episodes may account for up to 90% of the total energy expense of the hibernating period, and therefore is thought to underlie an essential function for the survival of the organism. In accordance with this scenario, hibernation in black bears, which is characterized by a continuous torpor but relatively high body temperature, is associated with the formation of permanent P-Tau aggregations in old animals [137]. In AD and other tauopathies, the constant high level of P-Tau coupled with a progressive increase in Ca^2+^-signaling alterations over the course of several years would lead to the formation of NFTs and ultimately cell death. Then, the decrease in CBP detection in the cortex interneurons during the development of AD could speed up the process with a positive feedback mechanism. In this respect, understanding what makes tau phosphorylation reversible in hibernating animals could lead to fundamental advancements in our understanding of AD pathogenesis and open the way to new studies aimed at recreating the same conditions, making hibernation a promising model in translational medicine.

## 6. Conclusions and Future Directions

We still have much to learn about animal hibernation, especially regarding how the integration of different molecular and biochemical mechanisms facilitates a precise and harmless transition to and from hypometabolic states. Particular attention should be given to CBPs due to their roles in cell activities and because several studies have hinted at the numerous processes specific to this annual cycle phase that are influenced by them. The variations observed in the expression and localization of buffer and sensor CBPs are thought to affect the activity of kinases and phosphatases which consequently exert neuroprotective functions by regulating ionic balance and suppressing cell degeneration and death. Then, these mechanisms lead to unmatched tolerance to severe conditions that could potentially be exploited to tackle several human pathologies, including neurodegenerative diseases. That some of these changes can be found in all hibernating animals raises the captivating possibility of independently evolved strategies that arose from common and crucial aspects of cell resistance and neuroprotection. Several works have also found a correlation between CBPs and the changes in cytoskeletal proteins between active and inactive stages, which seems to indicate an essential and conserved role for CBPs in neural plasticity. 

Since hibernation is a widespread adaptation, vertebrate and invertebrate models can be combined and compared, exploiting the advantages of each of them. In particular, mammals are more directly comparable with humans; their neuroanatomy is well known, and the roles of CBPs in normal and pathological conditions are established. On the other hand, invertebrate models can be used for the size of their neurons, the simplicity of their neural circuits, and the potential to correlate molecules and behaviors [223]. Finally, in light of the potential applications, numerous studies have tried to induce the hibernation-like state in non-hibernating or cellular models to exploit their amenability to numerous techniques and, for the latter, the possibility to study changes in the localization of various molecules within the cell [108,174,224,225]. One of the main limitations of these studies up until now is that it is difficult to distinguish real hibernation-induced protective strategies from passive reactions to hypothermia, hypoxia, or hypometabolism. A more comprehensive understanding of the processes that guide hibernation and the interactions between the protective strategies is needed before effectively using these models.

The information obtained up to this point on CBPs and hibernation opens exciting opportunities for both basic and applied research, and suggests that hibernation still has a lot to tell us about how cells work and adapt to different environmental conditions.

## Figures and Tables

**Table 1:** Changes in CBPs and cytoskeletal proteins during hibernation in vertebrates and invertebrates.

Species	Calcium-Binding Proteins				Cytoskeletal Proteins			Area	Ref.
	CB	PV	CR	CaM	MAP-2	P-TAU	NF-H		
*Ictidomys tridecemlineatus*	No change	No change	Decrease No change	Decrease Decrease	No change		Increase No change	Cortex Hypothalamus	[128]
							Increase (P-NP)	Forebrain	[122]
*Spermophilus citellus*					Decrease	Increase		Brain	[134]
						Increase		Forebrain	[135]
*Spermophilus lateralis*					Decrease			Brain (synapses)	[136]
*Spermophilus parryii*						Increase		Brain	[137]
*Mesocricetus auratus*						Increase		Forebrain	[138]
						Increase		Brain	[137]
						Increase		Brain	[71]
						Increase		Hippocampus	[139]
						Increase		Brain	[121]
*Erinaceus europaeus*	Decrease	Decrease	Decrease		Decrease		Decrease (P)	Cerebellum	[127]
*Ursus americanus*						Increase		Brain	[137]
*Rana esculenta*				Decrease No change	Decrease (f) Decrease		Decrease (P) Decrease (P)	Cerebellum Optic tectum	[126]
*Cornu aspersum*				Increase	Decrease (f)		Increase (P)	Cerebral ganglia	[95]
		No change		Increase	Increase	Increase	Increase (P-NP)	Cerebral ganglia	[133]
				Increase			Increase (P-NP)	Suboesophageal Ganglion	This work

CB: calbindin, PV: parvalbumin, CR: calretinin, CaM: calmodulin, P-TAU: high phosphorylated tau, NF-H: heavy subunit of neurofilaments.

**Table 2:** Effect of platinum compounds treatment on CBPs localization and NF-H phosphorylation in the rat CNS. CisPt: cisplatin (CisPt), PtAcacDMS: [Pt(*O,O′*-acac)(-acac)(DMS)].

Calcium-Binding Proteins		pNF-H	Compound	Area	Reference
CB	PV				
Decrease	Decrease	Increase	CisPt	Cerebellum	[18]
Decrease	Decrease	Increase	CisPt	Cerebellum	[188]
Decrease Decrease + no change			CisPt PtAcacDMS	Hippocampus	[186]
Decrease No change			CisPt PtAcacDMS	Cerebellum	[29]
	Decrease		CisPt	Cerebellum	[39]

**Table 3:** Changes in the detection of CBPs associated with age and Alzheimer’s disease in different mammal models.

**Age**						
**Species**	**CB**	**PV**	**CaM**	**Method**	**Area**	**Reference**
*Homo sapiens*	Decrease	No effect		CC	Cortex, Hippocampus	[200]
	Decrease	No effect	Decrease	tot mRNA	Frontal cortex	[197]
*Mus musculus*	Decrease	No effect		CC, WB	Striatum	[31]
	Decrease	No effect		CC, WB	Somatosensory cortex	[30]
		Decrease No effect		SI	Hippocampus (CA1) Hippocampus (CA3, DG)	[38]
		Decrease		CC, SI	Brain	[201]
			No effect Increase Decrease	RIA	Striatum Cortex, Cerebellum Diencephalon, Medulla	[196]
*Rattus norvegicus*	Decrease No effect			WB, ID	Hippocampus Cerebellum	[202]
	Decrease			WB	Striatum	[28]
	Decrease			CC, WB	Auditory system	[203]
	Decrease	No effect		CC, WB	Striatum	[31]
	Decrease	Increase		CC	Somatosensory cortex	[30]
*Meriones unguiculatus*	Decrease			SI, WB	Hippocampus	[204]
	Decrease	No effect		CC, WB	Striatum	[31]
	Decrease	Increase		CC, WB	Somatosensory cortex	[30]
*Mesocricetus auratus*	Decrease	No effect		ISH, ARG	Hippocampus, Striatum, Cerebellum	[205]
**Alzheimer’s Disease**						
**Species**	**CB**	**PV**	**CaM**	**Method**	**Area**	**Reference**
*Homo sapiens*	Decrease		Decrease	RIA	Frontal, Temporal, Parietal Cortex	[206]
	Decrease			CC, SI	Cortex	[207,208]
	Decrease			CC	Prefrontal cortex	[209]
	Decrease			CC	Temporal cortex	[210]
	Decrease			CC	Hippocampus	[195]
		Decrease		CC	Temporal, Parahippocampal, Parietal cortex, Cerebellum	[211]
		Decrease No effect		CC	Hippocampus	[212]
			Decrease	SI	Cortex	[213]
*Mus musculus*	Decrease			SI, WB, RT-PCR	Dentate gyrus	[214]
	Decrease			CC, SI	Hippocampus	[215]
		Decrease		SI, WB	Hippocampus	[216]

CC: cell count; WB: Western blot, SI: signal intensity; RIA: radioimmunoassay; ID: immunodot, ISH: in situ hybridization, ARG: autoradiography, CA1: *cornu Ammonis* 1, CA2: *cornu Ammonis* 2, DG: dentate gyrus.

## Abbreviations

AD	Alzheimer’s disease
ARG	Autoradiography
CA1, 2	*Cornu Ammonis* 1, 2
CaM	Calmodulin
CAMK	Ca^2+^/calmodulin-dependent kinase
CAMKII	Ca^2+^/calmodulin-dependent kinase 2
CB	Calbindin
CBPs	Calcium-binding proteins
CC	Cell count
CisPt	Cisplatin
CNS	Central nervous system
CR	Calretinin
DADLE	[D-Ala2, D-Leu5]-enkephalin
DG	Dentate gyrus
ERK1	Extracellular regulated protein kinase 1
ERK2	Extracellular regulated protein kinase 2
GABA	γ-aminobutyric acid
HIT	Hibernation induction trigger
HP	Hibernation-specific protein
ID	Immunodot
IHC	Immunohistochemistry
ISH	In situ hybridization
MAP	Microtubule-associated protein
MAP-2	Microtubule-associated protein 2
NF-H	Heavy subunit of neurofilaments
NFT	Neurofibrillary tangles
P	Phosphorylated
P-NP	Phosphorylated and non-phosphorylated
P-Tau	Phosphorylated tau
PKA	Protein kinase A
pNF-H	Phosphorylated heavy subunit of neurofilaments
PP1	Protein phosphatase 1
PP2A	Protein phosphatase 2A
PP2C	Protein phosphatase 2C
PtAcacDMS	[Pt(*O,O′*-acac)(γ-acac)(DMS)]
PV	Parvalbumin
RIA	Radioimmunoassay
SAPK/JNK	Stress-activated protein kinase/Jun-amino-terminal kinase
SI	Signal intensity
WB	Western blot
